# Diagnostic Performance of Prothrombin Time and Activated Partial Thromboplastin Time in Children: A Service Evaluation

**DOI:** 10.1111/ijlh.14557

**Published:** 2025-09-09

**Authors:** Gerard Gurumurthy, Mikias Lemma, Lianna Reynolds, John Grainger, Jecko Thachil

**Affiliations:** ^1^ The University of Manchester Manchester UK; ^2^ Department of Paediatric Haematology Royal Manchester Children's Hospital Manchester UK; ^3^ MAHSC Professor The University of Manchester Manchester UK

## Abstract

**Background:**

Coagulation screening, consisting of prothrombin time (PT) and activated partial prothrombin time (aPTT), is routinely performed in paediatrics to identify bleeding disorders or guide peri‐procedural management. We evaluated the trends in utilisation and diagnostic yield of PT and aPTT testing as part of coagulation screening in a tertiary paediatric centre.

**Methods:**

All PT and aPTT samples received from June to September 2024 were analysed. Total requests, sample rejection rates, abnormal result patterns (isolated PT, isolated APTT, combined), and clinical correlations were recorded. Laboratory cutoffs were PT > 12.5 s and APTT > 30.0 s. Youden's Index determined cutoffs associated with inherited bleeding disorders.

**Results:**

A total of 2808 coagulation profiles from 1207 patients were received, with 15.7% (442/2808) rejected in 268 patients. Of these, 31.7% (85/268) of patients were not re‐tested. Among valid requests, 17.0% (402/2366) were abnormal (128 isolated APTT, 173 isolated PT, 101 combined). In a subgroup of 337 randomly selected patients, 28.8% (97/337) had deranged results, leading to 12 new haematological and 34 acute diagnoses. Youden's index determined isolated APTT > 31.4 s associated with inherited disorders (AUC > 0.8). The same was not identified with isolated PT (PT > 13.0 s, AUC < 0.6).

**Conclusion:**

A substantial proportion of samples received are rejected, and some abnormal results remain unaddressed. Most abnormal findings are clinically significant, particularly when APTT > 33.1 s. There is scope to refine utilisation in paediatric practice.

## 1 Introduction

Coagulation screening assays, notably prothrombin time (PT) and activated partial thromboplastin time (aPTT), are useful for the assessment of haemostatic function [1, 2]. These assays detect abnormalities in both the extrinsic and intrinsic pathways, guiding clinicians in the diagnosis of inherited and acquired coagulopathy and the monitoring of anticoagulant therapy. However, these assays evaluate only discrete segments of the haemostatic cascade and lack the sensitivity to detect many bleeding disorders [3]. Furthermore, in vitro coagulation tests are inherently limited in their capacity to reproduce the complex physiological conditions under which clot formation occurs in vivo. The utility and limitations of routine PT and aPTT screening have been explored in adults previously. Several studies question the value of indiscriminate testing in asymptomatic individuals and instead advocate for targeted, history‐driven approaches [4, 5, 6].

In paediatric practice, however, comprehensive data regarding the performance characteristics, optimal thresholds, and clinical yield of PT and aPTT remain limited. Phlebotomy in children poses unique challenges. These include smaller blood volumes, difficulty with venous access, and a higher likelihood of pre‐analytical errors such as venipuncture‐related stress, activated samples and underfilled samples [7, 8, 9]. These factors may contribute to elevated sample rejection rates and impact diagnostic accuracy. Moreover, the physiology of the haemostatic system differs in neonates and children, leading to different normal ranges for PT and aPTT [8, 9]. It is also notable that the relative prevalence and spectrum of coagulation disorders differ between paediatric and adult cohorts; a coagulation screen is more likely to identify a previously unknown congenital factor deficiency in children as well as vitamin K‐dependent anomalies related to nutritional status and illness [10, 11, 12].

Given these considerations, there is a need to characterise real‐world patterns of PT and aPTT utilisation, assess the integrity of collected samples, and determine the diagnostic value of abnormal results in a paediatric setting.

## 2 Methods

### 2.1 Study Design and Patient Selection

A retrospective study was conducted at a tertiary referral centre. All PT and aPTT processed that were received between 1 June 2024 and 30 September 2024 were included from patients < 18 years old. Ethics approval was not required as this was a service evaluation.

### 2.2 Data Sources and Collection

Laboratory information system records were interrogated to extract patient identifiers, age, sex, requesting specialty, date and time of sample collection, and assay results. Electronic health records were subsequently reviewed for all cases with abnormal coagulation times to ascertain follow‐up investigations, specialist referrals and definitive diagnoses.

### 2.3 Laboratory Procedures

Venous blood was drawn into sodium citrate tubes at a 9:1 ratio. All samples were centrifuged within 1 h of collection. Following centrifugation, any sample with visible haemolysis, clotting, insufficient volume (< 10% below the fill line for adults; exactly to the line for children) or other pre‐analytical errors was rejected.

PT and aPTT coagulation assays were performed on the Sysmex CN‐Series analysers (Sysmex Corporation, Kobe, Japan), which measure transmitted light through the plasma‐reagent mixture at five LED wavelengths (340, 405, 575, 660 and 800 nm) via an optical fibre and photodiode detector. Clotting times and kinetic parameters were computed by on‐board microprocessors, and all PT and aPTT assays share the same optical and cuvette modules. Quality control measures are strictly adhered to, including calibration with manufacturer‐provided reagent and automated sample assessment prior to each run and after any QC failure, in line with the CN‐Series Calibration and Batch Acceptance SOP. Finally, all validated results were transmitted to the LIMS for clinical reporting.

Institutional reference ranges were PT 9.5–12.5 s and APTT 25.0–30.0 s for children > 1 year old. For children < 1 year old, reference ranges varied and were assessed using normal ranges adjusted by age and gestation as per local Paediatric Haematology advice. Results exceeding these upper limits were flagged as prolonged. Further investigations of abnormal PT/aPTT are ordered by the requesting clinicians if appropriate and in consultation with haematology by that team. This may lead to additional tests including clotting factor assays and/or inhibitors.

### 2.4 Definition of Outcome Measures

The primary outcomes were [1] sample rejection rate and [2] the proportion of valid samples with isolated PT prolongation, isolated APTT prolongation, or combined prolongation. Secondary outcomes included the proportion of abnormal results leading to clinically significant diagnoses, stratified by haematological versus non‐haematological aetiologies in a subset of patients. Clinically significant diagnoses were defined as new or progressive disorders necessitating therapeutic intervention or specialist follow‐up.

### 2.5 Subgroup Analysis

A randomly selected subgroup of patients with any abnormal coagulation result underwent detailed chart review. Further testing was done based off the PT and aPTT results were instigated by the treating team. We serial numbered all patients from 1 through 1207 and then computer‐generated random serial numbers of patients for further chart review. We reviewed the derived diagnoses associated with the deranged coagulation screening. We selected a precision‐based sample size using the ‘worst‐case planning prevalence’ (*p* = 0.50), which maximises variance. We used a precision‐based sample size with the worst‐case planning prevalence (*p* = 0.50), which maximises *p*(1 − *p*) and thus yields the largest required sample. This conservative assumption ensures adequate precision irrespective of the true prevalence. With 95% confidence and ±5% precision, and applying the finite‐population correction for a sampling frame of the total number of patients (*n* = 1207), the required sample was determined to be 292 patients. We exceeded this threshold during study duration and chart‐reviewed 337 individuals.

### 2.6 Statistical Analysis

Descriptive statistics summarised demographic and laboratory data. Comparisons between subgroups employed chi‐square or Fisher's exact tests for categorical data and *t*‐tests or Mann–Whitney U tests for continuous variables. *p*‐values < 0.05 deemed significant. Receiver Operating Characteristic (ROC) curves for PT and aPTT in detecting inherited bleeding disorders were constructed, with area under the curve (AUC) calculated. Optimal cut‐off values were determined by maximising Youden's index (sensitivity + specificity−1). AUC values ≥ 0.80 were considered indicative of good discriminative performance. All analyses were performed using R version 4.2.

## 3 Results

### 3.1 Baseline Characteristics

During the review period, 2808 PT and aPTT profiles were received for 1207 unique paediatric patients. The majority were male (676/1207, 56.0%) The median age of the cohort was 6.4 years (IQR 2.1–12.7), with 8.6% (104/1207) less than 1 year old.

### 3.2 Sample Requests and Rejection

Intensive care units (ICU) accounted for the largest share of samples received (841/2808, 30.0%), followed by general paediatric wards (620/2808, 22.1%), and emergency department (550/2808, 19.6%). Haematology clinics (67/2808, 2.4%) and various surgical specialties (36/2808, 1.3%) accounted for a smaller proportion.

Of all submitted samples, 442/2808 (15.7%) were rejected. These rejections affected 268 patients, with 31.7% (85/268) failing to undergo repeat sampling. Rejection rates varied by setting, peaking in the emergency department (18.9%) and lowest in haematology clinics (6.0%).

### 3.3 Distribution of Abnormal Coagulation Profiles

Among the 2366 valid assays, 402 (17.0%) demonstrated prolongation: isolated aPTT in 128 cases (5.4%), isolated PT in 173 cases (7.3%), and combined prolongation in 101 cases (4.3%). Age‐based analysis showed no significant difference in abnormal clotting rates between patients < 1 year (34.6%) and those ≥ 1 year (32.4%, *p* > 0.05). The ICU cohort exhibited the highest abnormality rate (265/841, 31.5%). Haematology clinics and surgical specialties reported abnormal rates of 22.4% (15/67) and 11.1% (4/36), respectively. The median PT among prolonged results was 14.2 s (IQR 13.1–16.8) and median aPTT 38.5 s (IQR 33.2–45.7).

### 3.4 Subgroup Analysis, Clinical Correlates and Diagnostic Yield

**FIGURE 1:**
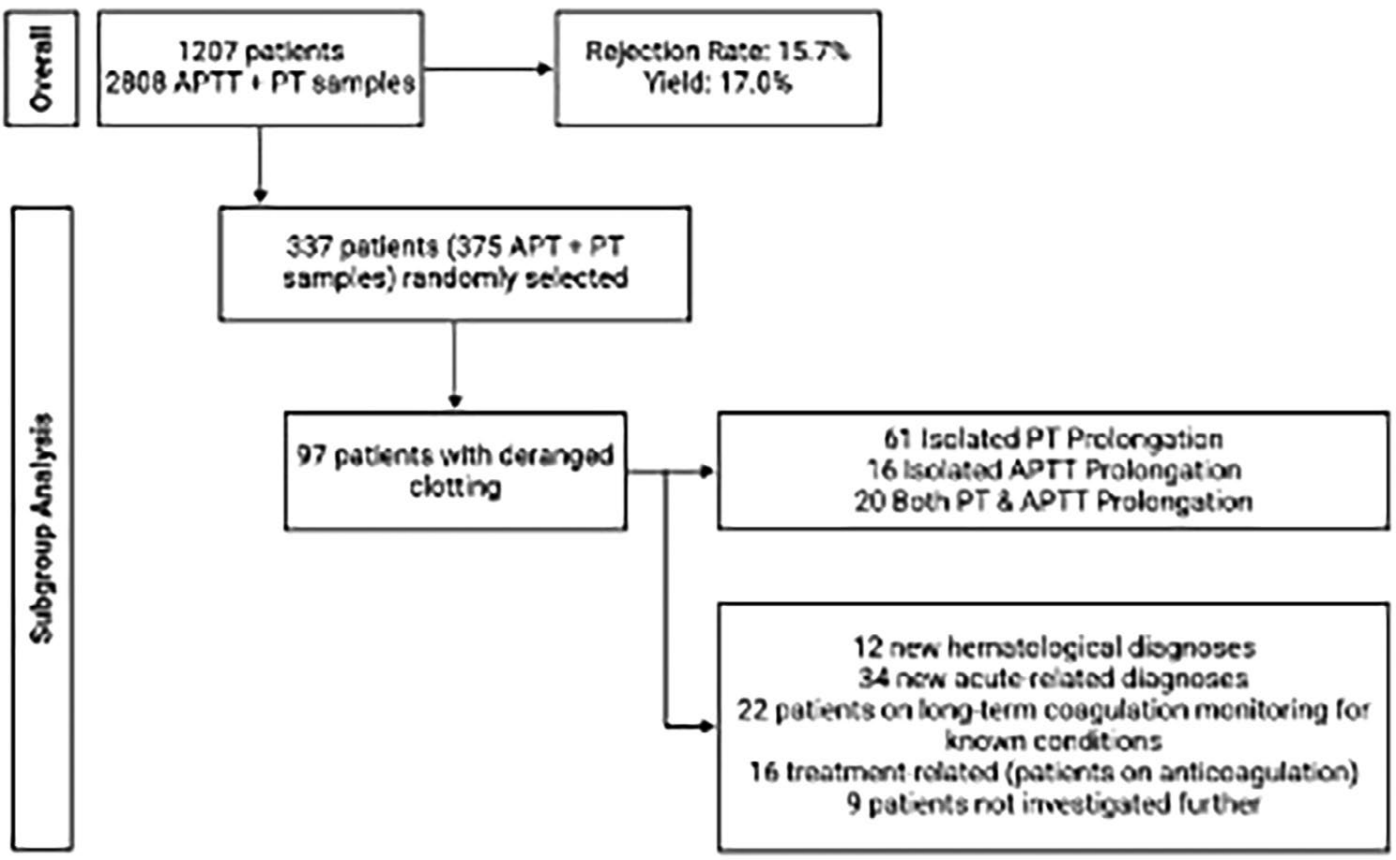
Flowchart of PT/aPTT testing and subgroup analysis. Among 1207 paediatric patients, 2808 PT/APTT samples were submitted, with 15.7% rejected and 17.0% yielding abnormal results. For detailed chart review, 337 patients (375 samples) were randomly selected; 97 of these patients demonstrated deranged clotting (61 isolated PT prolongation, 16 isolated APTT prolongation, 20 combined prolongation). Clinical follow‐up in this subgroup revealed 12 new haematological diagnoses, 34 new acute non‐haematological diagnoses, 22 patients on long‐term monitoring for known coagulation disorders, 16 treatment‐related abnormalities (anticoagulation) and 9 cases not investigated further.

A total of 337 patients with any abnormal result underwent detailed chart review in our subgroup analysis (Figure 1). Over 87% of isolated PT, isolated APTT, and combined prolongations were deemed clinically actionable, prompting further specialist evaluation. Among these, 97/337 (28.8%) with deranged coagulation results received new clinically significant diagnoses from further investigations: 12 haematological disorders (6 factor VIII or IX deficiencies, 3 acute leukaemias, 2 von Willebrand diseases, 1 factor XI deficiency) and 34 acute non‐haematological conditions (9 infections, 7 sepsis‐induced coagulopathy, 3 traumatic injuries, 2 adrenal crises, 2 trauma related intracranial haemorrhages, 2 toxic ingestions, 1 acute liver failure, 1 renal cortical necrosis) (Table 1). Previously known disorders were identified in 19 patients (6 haematological, 13 non‐haematological, predominantly renal and hepatic).

**TABLE 1:** Clinical diagnoses identified in the subgroup of 97/337 paediatric patients with abnormal PT/aPTT results.

Diagnoses from subgroup cohort	*N* = 97
New haematological diagnoses	
Clotting Factor Deficiencies	6 (6.2%)
Malignancies	3 (3.1%)
Von Willebrand Disease	2 (2.1%)
Inherited bleeding disorders	1 (1.03%)
Previously known haematological diagnoses on follow‐up	
Clotting Factor Deficiencies	3 (3.1%)
Von Willebrand Disease	2 (2.1%)
Malignancies	1 (1.03%)
Non‐haematological acute diagnoses	
Infections	9 (9.3%)
Sepsis‐induced coagulopathy	7 (7.2%)
Trauma	3 (3.1%)
Endocrine	2 (2.1%)
Neurological	2 (2.1%)
Toxicological	2 (2.1%)
Hepatic	1 (1.03%)
Renal	1 (1.03%)
Previously known non‐haematological diagnoses on follow‐up	
Renal	5 (5.2%)
Hepatic	3 (3.1%)
Inherited	3 (3.1%)
Others	2 (2.1%)

### 3.5 ROC Curve Analysis

In detecting inherited bleeding disorders (*n* = 9 confirmed cases), aPTT demonstrated strong discriminative ability (AUC = 0.82, 95% CI 0.75–0.88), with an optimal threshold at 31.4 s (Youden's index J = 0.72; sensitivity 0.81, specificity 0.91). In contrast, PT exhibited poor performance (AUC = 0.58, 95% CI 0.50–0.66) and failed to yield a clinically useful cut‐off.

## 4 Discussion

This report on PT and aPTT utilisation highlights significant pre‐analytical, as well as the considerable clinical impact of abnormal coagulation findings. We report a pre‐analytical error of 15.7% as rejected coagulation samples. In comparison to adults/mixed cohorts, it is thought that the pre‐analytical error of laboratory tests in general is around 60% [13, 14]. We note that a substantial number of patients with rejected samples failed to undergo repeat testing, suggesting injudicious use of the resource initially. Elevated rejection rates in emergency settings suggest opportunities for targeted phlebotomy training and protocol reinforcement. Lower rejection rates in haematology clinics indicate that such training can improve rejection rates. This, in turn, has been associated with lower cost through laboratory expenditure and in‐hospital stay [15, 16, 17]. Repeated sampling is also associated with the risk of iatrogenic anaemia [18].

The ICU's disproportionately high yield of 31.5% is anticipated in the context of critical illness, where systemic inflammatory responses induce widespread activation of coagulation cascades via tissue factor expression and consumptive coagulopathy [19]. In septic shock and trauma, endothelial cell activation and glycocalyx degradation compromise vascular integrity, promoting microvascular thrombosis and contributing to prolonged clotting times [20, 21]. Acute liver dysfunction in multi‐organ failure further prolongs PT/aPTT through reduced synthesis and impaired clearance of clotting factors [21, 22, 23]. It is therefore understandable that the ICU is also associated with the largest share of requests at 30.0%.

Importantly, our subgroup analysis revealed that 28.8% of patients with abnormal tests received new clinically important diagnoses directly attributable to the test finding. The finding suggests a greater diagnostic yield in paediatrics, where the pre‐test probability of new congenital disorders is higher. ROC analysis confirms that APTT prolongation > 31.4 s offers strong discrimination for intrinsic pathway deficiencies. However, incidental prolongation of aPTT in children is common, and a repeat screening is warranted before consideration for further investigations [24]. Conversely, PT's poor sensitivity limits its utility as a standalone screen for inherited bleeding disorders [25]. This is a finding determined by our ROC analysis, where PT was a poor discriminant (AUC = 0.58). It does, however, remain critical for monitoring vitamin K‐dependent factor dysfunction in liver disease and warfarin therapy [26].

In our cohort, surgical specialties accounted for a small subset of all PT/aPTT requests and demonstrated a low yield at 11.1%. This low diagnostic yield mirrors the British Society for Haematology guidance. It advises against indiscriminate pre‐operative coagulation screening in unselected patients, noting that routine PT/aPTT testing neither predicts bleeding risk nor does a normal result exclude a bleeding disorder [5]. Instead, these guidelines recommend a structured bleeding history, encompassing personal and family bleeding events and medication review, as the primary tool for peri‐operative risk assessment. The guidance is reported for adults, with implications for neonates and young children not known. However, by reserving a coagulation screen for those with a positive history or clear clinical indication, excess costs to patients and services could be avoided.

We therefore recommend that PT and aPTT should not be ordered as part of routine pre‐operative panels or in patients without any personal or family bleeding history. Instead, they should be reserved for:

1. those with a positive bleeding questionnaire,
2. children on anticoagulant or hepatotoxic therapy and
3. critically unwell patients in whom acquired coagulopathy is suspected (e.g., sepsis, trauma, liver failure).

When ordered, aPTT prolongation (> 31.4 s in our report) alongside a bleeding history should trigger a haemostasis work‐up as it may identify intrinsic pathway deficiencies with high sensitivity and specificity. PT prolongation, in our report, is less discriminatory for congenital bleeding disorders and may instead direct attention to vitamin K status, liver function, disseminated intravascular coagulation, or anticoagulant effects. In those contexts, actionable thresholds will vary. Any value above the local upper limit merits clinical correlation, vitamin K assessment, and, where appropriate, specialist input. Overall, judicious test ordering based on history, together with clear cut‐off driven follow‐up pathways, will maximise diagnostic yield and minimise the financial and patient costs of unnecessary investigations.

Our service evaluation has several limitations. First, it was a single‐centre, retrospective analysis and is therefore susceptible to information bias and unmeasured confounding. Second, our population was heterogeneous (ICU, inpatient, emergency, outpatient), and we did not power or stratify the study to detect differences within each clinical setting. The surgical cohort was small, limiting conclusions regarding pre‐procedural screening in that subgroup. Third, only a randomly selected subgroup (337/1207; ~28%) underwent detailed chart review. This introduces verification/spectrum bias as patients with normal results outside the audited subgroup may have had undetected disorders, and patients with abnormal results may have been more intensively investigated. Lastly, our ROC analysis for inherited bleeding disorders was derived from the chart‐reviewed subgroup and based on a small number of confirmed cases (*n* = 9). The resulting AUC estimates and Youden thresholds must be analysed cautiously. Moreover, aPTT thresholds and diagnostic performance are reagent/analyser‐specific (Sysmex CN‐series with local reagents). This limits the generalizability of the cut‐off values derived. Small pre‐analytical or analytical shifts around the upper reference limit may alter classification.

## 5 Conclusion

Our service evaluation examines real world data identifying that a substantial number of aPTT and PT samples received are rendered invalid by sample quality issues and that a large proportion of these were not repeated. Importantly, the vast majority of detected abnormalities are clinically significant, particularly with an isolated prolonged aPTT. These findings underscore the need to refine paediatric coagulation screening by discernment regarding when to collect samples and improving phlebotomy practices to reduce sample rejection, followed by prompt action. This strategy would maximise diagnostic yield and minimise unnecessary investigations.

## Author Contributions

**Gerard Gurumurthy:** writing – original draft, data collection, data analysis. **Mikias Lemma:** writing – original draft, data collection, data analysis. **Lianna Reynolds:** data analysis, writing – review and editing. **John Grainger:** writing – review and editing. **Jecko Thachil:** writing – review and editing, conceptualisation.

## Ethics Statement

The authors have nothing to report.

## Consent

The authors have nothing to report.

## Conflicts of Interest

The authors declare no conflicts of interest.

## Data Availability

The data that support the findings of this study are available on request from the corresponding author. The data are not publicly available due to privacy or ethical restrictions.
